# Projected Outcomes of Removing Fluoride From US Public Water Systems

**DOI:** 10.1001/jamahealthforum.2025.1166

**Published:** 2025-05-30

**Authors:** Sung Eun Choi, Lisa Simon

**Affiliations:** 1 Department of Oral Health Policy and Epidemiology, Harvard School of Dental Medicine, Boston, Massachusetts; 2 Division of General Internal Medicine, Brigham and Women’s Hospital, Boston, Massachusetts

## Abstract

**Importance:**

Fluoridation of public water systems has been a cornerstone of public health efforts in the US but has come under increasing scrutiny due to concerns of neurotoxicity.

**Objective:**

To determine the cost-effectiveness of cessation of public water fluoridation and associations with oral health outcomes among US children.

**Design, Setting, and Participants:**

A cost-effectiveness analysis was conducted to estimate changes in total tooth decay, quality-adjusted life-years (QALYs), and costs associated with removal of fluoride in public water system during 5- and 10-year periods. A microsimulation model of oral health outcomes was constructed based on oral health and water fluoridation data of US children from age 0 to 19 years in a nationally representative sample from the US National Health and Nutrition Examination Survey (NHANES), 2013 to 2016. Sensitivity analyses were conducted to assess the robustness of the simulation results to variation in model input parameters. Data analysis was conducted from November 15, 2024, to February 3, 2025.

**Exposure:**

Cessation of public water fluoridation in the US.

**Main Outcomes:**

Changes in dental caries prevalence; total number of decayed teeth and dental fluorosis; QALYs; and costs.

**Results:**

The simulation model was informed by NHANES data of 8484 participants (mean [SD] age, 9.6 (0.1) years; 4188 [weighted percentage, 49.0%] female). In the base-case scenario of eliminating fluoridation, dental caries prevalence and total number of decayed teeth were estimated to increase by 7.5 (95% uncertainty interval [UI], 6.3 to 8.5) percentage points and by 25.4 million (95% UI, 23.3-27.6 million) cases, with a loss of 2.9 million (95% UI, −3.2 to −2.6 million) QALYs at a cost of $9.8 billion (95% UI, $8.7 to $10.8 billion) over 5 years. Sensitivity analyses estimating less efficacy from fluoridation found lower but still substantial harms. Estimates increased for a 10-year horizon compared to a 5-year horizon. Increased tooth decay would disproportionately affect publicly insured and uninsured children compared to those with private dental insurance.

**Conclusions and Relevance:**

This cost-effectiveness analysis found that cessation of public water fluoridation would increase tooth decay and health system costs in the US. Despite concerns regarding toxic effects associated with high levels of fluoride, this model demonstrates the substantial ongoing benefits of water fluoridation at safe levels currently recommended by the US Environmental Protection Agency, the National Toxicity Program, and the Centers for Disease Control and Prevention.

## Key Points

**Question:**

What are the projected outcomes of ceasing to fluoridate public water on rates of tooth decay and the cost of dental care among children in the US?

**Findings:**

This cost-effectiveness analysis using data for 8484 children (mean age, 9.6 years) from the US National Health and Nutrition Examination Survey for 2013 to 2016 found that elimination of fluoride would be associated with an increase in tooth decay of 7.5 percentage points and cost approximately $9.8 billion over 5 years.

**Meaning:**

Cessation of fluoridation of US public water systems is projected to worsen oral health in children and to significantly increase national health care costs.

## Teaser

This cost-effectiveness analysis evaluates a large sample of US children to determine the associations of fluoridation cessation with oral health and national dental care costs.

## Introduction

Since it was first implemented in Grand Rapids, Michigan, in 1945, fluoridation of public water systems (PWS) in the US has been hailed as a major public health victory that reduces tooth decay.^1^ Fluoride prevents tooth decay through 2 mechanisms: by converting hydroxyapatite in tooth enamel to the more acid-resistant fluorhydroxyapatite and by inhibiting some bacterial enzymes.^2^

Excessive fluoride exposure can cause mottled discoloration of the teeth (ie, fluorosis) and, more critically, becomes a neurotoxin at high levels.^3^ Natural sources of drinking water with high levels of fluoride (eg, due to groundwater absorption) are associated with lower IQ scores.^4,5^

For this reason, fluoridation of PWS has come under fire.^6^ The US National Toxicology Program released a monograph^7^ and related meta-analysis^8^ that concluded that drinking water with elevated fluoride levels has neurotoxic effects, but affirmed a lack of evidence for neurocognitive effects with fluoride exposure less than 1.5 parts per million, more than twice the amount of fluoridation recommended in PWS by the US Centers for Disease Control and Prevention.^9^ Several studies have found that prenatal maternal exposure to fluoride, even at recommended levels, may be associated with behavioral challenges in young children,^10,11^ although the methods used by these studies have been challenged,^12,13^ and other studies have not found this association.^14,15,16^ The Secretary of the Department of Health and Human Services, Robert F. Kennedy Jr, has pledged to remove fluoride from the US water supply.^17^

Yet, the US Preventive Services Task Force provides a B grade for application of topical fluoride varnish for all children and fluoride supplementation for children who are drinking unfluoridated water.^18^ Evidence has demonstrated increased dental disease, and subsequent harms, when fluoridation is eliminated^19,20^; and fewer caries in children exposed to fluoridated PWS.^21^ In Calgary (Alberta, Canada) fluoridation was reintroduced to the PWS as of March 2025 in response to the rise in dental disease following its removal in 2011.^22^

The purpose of this study was to estimate how cessation of water fluoridation would affect the dental health and health care costs of US children by conducting model-based economic evaluation. Because there is no consensus on the neurocognitive effects of fluoride at the levels used in PWS and current federal guidance does not find an association between PWS fluoridation and neurocognitive decline, we did not include this outcome in the model.

## Methods

This study was reviewed by the institutional review board of the Harvard Medical School and was determined to be exempt from the requirement of approval and from informed consent because the study used only deidentified data. We followed the International Society for Pharmacoeconomics and Outcomes Research (ISPOR) reporting guideline.

A cost-effectiveness analysis was conducted to examine how removal of fluoride in PWS would be expected to affect the risk of the dental caries (tooth decay) and associated quality-adjusted life years (QALYs) and costs. We did not include cognitive outcomes in our model due to a lack of data to support this impact at the fluoridation levels found in the PWS in the US. A stochastic microsimulation model of oral health outcomes using a decision analytic framework was developed and validated to account for variations in individual key traits across children residing in areas with different fluoride levels that may influence the impact of changes in fluoride levels in PWS (eMethods and eTables 1 to 4 in Supplement 1).

### Data Sources

Table 1 summarizes the key model parameters and data sources.^1,8,9,10,11,12,13,14,22,23,24,35,36,39,40^ Baseline demographic characteristics, dental utilization, oral health examination, and access to fluoride in PWS data were obtained from the National Health and Nutrition Examination Survey (NHANES; N = 8484 participants aged <20 years), 2013 to 2016. NHANES is the only national survey in the US that contains clinical oral health examination data rather than self-reported dental outcomes. Survey sample weights were used to correct for differential sampling and nonresponse in NHANES.^39,40^ Other model input parameters, such as effectiveness of water fluoridation on reducing the risk of tooth decay, were obtained from published peer-reviewed literature, further detailed in Table 1.

**Table 1.** Model Parameters for Determining Cost-Effectiveness of Discontinuing Fluoridation of US Public Water Systems

Parameter	Base-case value, % (range)	Distributional assumption	Source
Population characteristics, by fluoridated water level	eTable 2 in Supplement 1	NA	NHANES, 2013-2016
Effectiveness of water fluoridation on reducing tooth decay risk	25.0 [7.5-35.0]	β	Iheozor-Ejiofor et al^1^; Boehmer et al^23^
Disease risk			
Baseline dental caries	eTable 2 in Supplement 1	NA	NHANES, 2013-2016
Baseline dental utilization	eTable 3 in Supplement 1	NA	NHANES, 2013-2016
All-cause mortality rate	eMethods in Supplement 1	NA	CDC^24^
Risk of dental caries	Calibrated, model validation in eFigure in Supplement 1	NA	Model-based estimates
Probability of untreated caries	72.0 (45.8-72.0)	β	Fleming et al^25^; CDC^26^
Probability of tooth abscess for untreated caries	32.1 (30.0-46.4)	β	Azodo et al^27^; Srivastava^28^; Schnabl et al^29^
Probability of tooth loss for untreated caries	76.6 (66.3-85.5)	β	Monte-Santo et al^30^
Probability of moderate to severe fluorosis in excessively fluoridated areas	7.0 (7.0-12.0)	β	NHANES, 2013-2016
Disutility weights, mean (SD; range)			
Dental caries	0.010 (0.003; 0.004-0.019)	β	Kay et al^31^; IHME^32^
Tooth abscess	0.069 (0.015; 0.029-0.110)	β	Brennan et al^33^
Tooth loss	0.067 (0.013; 0.045-0.095)	β	IHME^32^; IHME^34^
Cost, mean (SD; range), $			Humana,^35^ADA,^36^ Atkins et al^37^
Examination	185 (10; 45-210)	γ	
Dental caries	530 (20; 325-977)	γ	
Tooth abscess	818 (45; 309-1220)	γ	
Tooth extraction	181 (10; 96-360)	γ	
Moderate to severe fluorosis	1468 (420; 1050-1850)	γ	
Water fluoridation, mean (SD; range)			
Annual per capita cost^a^	0.8 (3.5; 0.6-15.0)	γ	O’Connell et al^38^

Abbreviations: ADA, the American Dental Association; CDC, US Centers for Disease Control and Prevention; NA, not applicable; NCHS, National Center for Health Statistics; NHANES, National Health and Nutrition Examination Survey.

^a^ Annual per capita cost was calculated as a weighted average cost based on size of community served and associated costs available in a study by O’Connell et al^38^ using data from the American Dental Association.^36^

### Simulation Model

We simulated a nationally representative sample of 10 000 US children (age 0 to 19 years), starting in December 2024, to estimate changes in total costs, QALYs, and cumulative dental caries incidence with a removal of fluoride in the PWS accounting for differences in demographic composition, disease risks, and access to dental care across the populations residing in areas with different fluoride levels in PWS (eTable 1 in Supplement 1). We classified the synthetic population in this model by combinations of a few key demographic characteristics: age group (2-5, 6-12, or 13-19 years); sex (female or male); race and ethnicity per the NHANES self-identified survey response to the options: Hispanic (Mexican-American or other Hispanic), non-Hispanic Black, non-Hispanic White, or other (individuals who self-identified as belonging to other races or as multiracial and did not identify as Hispanic); income group (<130% of the federal poverty level [FPL], middle [130%-300% of FPL], and high [>300% of FPL]); health insurance type (private, public, or uninsured); and access to fluoride through the PWS by fluoride concentration level (below detection limit; less than optimal, 0.1 to ≤0.6 mg/L; optimal, 0.6 to ≤1.5 mg/L; or excessive, >1.5 mg/L).

The risk of developing new dental caries was estimated for each individual as a function of age, sex, race and ethnicity, and annual income. Binary indicators for caries incidence were assigned to each simulated individual and summed to calculate the total number of decayed teeth for the simulated individuals. To ensure validity of the model, we calibrated the model against dental caries prevalence from NHANES (dental caries being defined as having signs of decay, being filled on the crown or enamel surface of a tooth, or missing due to caries),^41^ by age groups and race and ethnicity (eFigure in Supplement 1).

We simulated 2 scenarios: (1) status quo, ie, maintaining the current fluoride levels in PWS; and (2) base-case scenario of reducing fluoride levels to 0 mg/L in all water systems. In the base-case scenario, it was assumed that individuals residing in areas with optimal fluoride levels (>0.6 mg/L) were receiving protective benefits from fluoridated water by reducing the risk of tooth decay, and individuals living areas with less than optimal levels (≤0.6 mg/L) were not assumed to receive any protective benefits from fluoride.^1,23^ Individuals residing in areas with excessive fluoride levels (>1.5 mg/L) were assumed to experience risk of developing moderate to severe dental fluorosis based on an analysis of NHANES data. The estimated outcomes of the simulation interventions included dental caries prevalence, cumulative caries incidence (total number of decayed teeth), cumulative moderate to severe fluorosis incidence (total number of fluorosis), and incremental QALYs and costs. The model was simulated over 5- and 10-year periods to be consistent with policy planning horizons, and to minimize longitudinal uncertainty in the estimates.

Costs and QALY estimates were integrated over the simulated period for all simulated individuals from a health care perspective. Treatment costs were obtained from the American Dental Association, claims data, and a prior cost-effectiveness analysis (Table 1).^35,36,37^ Disutility weights of disease states to calculate QALYs were based on large-scale survey data and prior cost-effectiveness analyses.^31,32,33^ Costs were expressed in 2024 US dollars using the Consumer Price Index,^42^ Personal Health Care Dental Service, and Personal Consumption Expenditure,^43^ and costs and QALYs were discounted at 3% annually.

### Sensitivity and Uncertainty Analyses

We performed a probabilistic sensitivity analysis by sampling from the probability distributions of all input parameters. The parameter ranges and distributions used in our sensitivity analyses are summarized in Table 1. Simulated individuals were re-run 1000 times with repeated Monte Carlo sampling from the probability distributions of all input parameters to capture uncertainties in our estimates, generating 95% uncertainty intervals (95% UIs) according to the reporting guidelines.^44,45^ In additional sensitivity analyses, we evaluated the impact of maintaining optimal fluoride levels in all currently fluoridated areas, thus expanding protective benefits from fluoridated water currently at levels of 0.1 to 0.6 mg/L. Moreover, while we assumed that all individuals residing in fluoridated areas received protective benefits from fluoride in the base-case scenario, we assessed the impact of limiting protective benefits to only those drinking tap water in these areas as a sensitivity analysis. Additional 1-way sensitivity analyses were performed to assess changes in the estimated outcomes across a wide range of values for 9 model parameters related to effectiveness of water fluoridation, risk of dental fluorosis, treatment cost, and disutility weights by setting individual parameters at their extreme values (eTable 4 in Supplement 1). Supplement 1 details all input data and complete technical details. All analyses were performed from November 15, 2024, to February 3, 2025, using R, version 4.4.1 (The R Foundation for Statistical Computing).

## Results

The simulated population was informed by NHANES data of 8484 participants (mean [SD] age, 9.6 [0.1] years; 4188 female [weighted percentage (wt%), 49.0] and 4296 male [wt%, 51.0]; 1979 Black [wt%, 13.8], 2848 Hispanic [wt%, 24.3], 2334 White [wt%, 51.6], and 1323 individuals of other race and ethnicity [wt%, 10.3]). If there were no changes to the current water fluoridation levels and health risk factor profiles, our model estimated that the dental caries prevalence would be 21.3% (95% CI, 18.6%- 24.0%) among children 2 to 5 years old; 51.6% (95% CI, 47.7%-54.8%) among those 6 to 12 years old; and 57.2% (95% CI, 54.9%-56.0%) among individuals 13 to 19 years old (eFigure in Supplement 1). Additional validation results show that model-predicted values of the status quo matched outcomes from the observed data within less than 5% absolute error (eFigure in Supplement 1).

**Figure 1.**
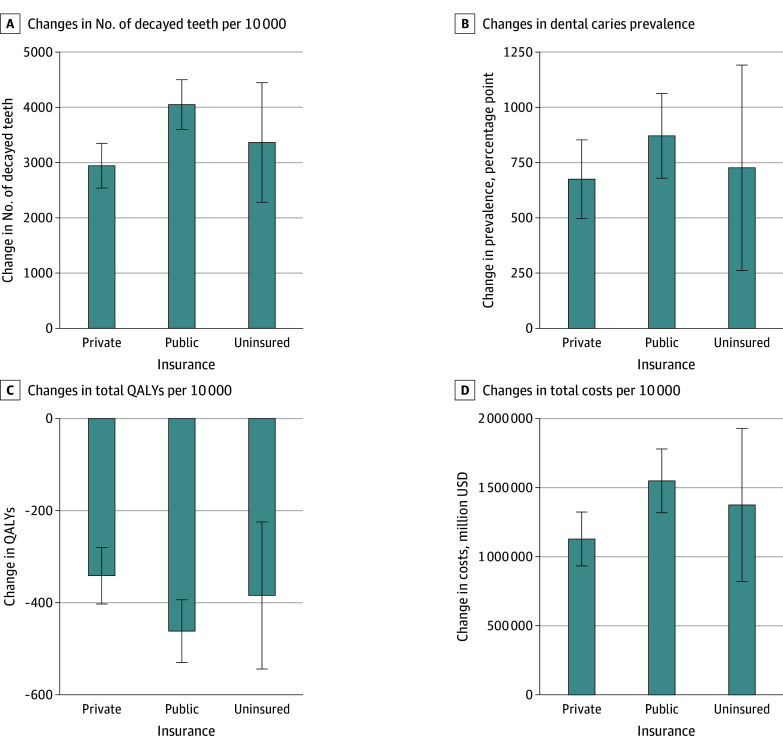
Simulated Outcomes of Discontinuing Fluoridation of the US Public Water System Over a 5-Year Period, by Insurance Status Error bars indicate 95% uncertainty intervals. QALYs indicates quality-adjusted life years.

In 2016, 40.4% of US children had access to optimal fluoride levels that effectively prevent tooth decay, while 45.7% had access to a less than optimal level and 1.5% had exposure to an excessive level (risking fluorosis or other harms) (eTable 1 in Supplement 1). If fluoride were removed from the PWS, the model estimated that dental caries prevalence and total decayed teeth would increase by 7.5 (95% UI, 6.3 to 8.5) percentage points (pp) and 25.4 million (95% UI, 23.3 to 27.6 million) teeth and decrease total number of fluorosis by 0.2 million (95% UI, −0.3 to −0.1 million) cases over a 5-year period (Table 2). Removing fluoride would cost $9.8 billion (95% UI, $8.7 to $10.8 billion), mainly due to increased risk of tooth decay and associated complications. After 10 years, the total number of decayed teeth would increase to 53.8 (95% UI, 50.6 to 57.0) at a cost of $19.4 billion (95% UI, $17.9 to $20.9 billion). These negative consequences in terms of health outcomes and costs accrued the most among publicly insured children given current distribution of access to fluoride through PWS across the US by insurance status (Figure 1).

**Table 2.** Cost-Effectiveness Results and Oral Health Outcomes Among Children in the US

Projection	Mean (95% uncertainty intervals)^a^				
	Total changes			Incremental changes	
	Dental caries prevalence, percentage points	Decayed teeth, No. in millions	Fluorosis cases, No. in millions	QALYs gained/lost, millions	Cost, $ billions
**Preventive benefits of fluoride among those living in fluoridated area**					
5-y Projection					
Removing fluoride	7.5 (6.3 to 8.5)	25.4 (23.3 to 27.6)	−0.2 (−0.3 to −0.1)	−2.9 (−3.2 to −2.6)	9.8 (8.7 to 10.8)
Optimize fluoride levels for suboptimal communities	−6.9 (−8.2 to −5.6)	−22.0 (−24.2 to −19.8)	−0.2 (−0.3 to −0.1)	2.6 (2.2 to 2.9)	−9.3 (−10.4 to −8.3)
10-y Projection					
Removing fluoride	7.6 (6.4 to 8.8)	53.8 (50.6 to 57.0)	−0.5 (−0.6 to −0.3)	−9.5 (−10.3 to −8,7)	19.4 (17.9 to 20.9)
Optimize fluoride levels for suboptimal communities	−7.7 (−9.0 to −6.5)	−49.7 (−53.0 to −46.4)	−0.5 (−0.6 to −0.3)	9.1 (8.3 to 9.8)	−19.6 (−21.2 to −18.1)
**Preventive benefits of fluoride among those drinking tap water in fluoridated area**					
5-y Projection					
Removing fluoride	6.0 (4.7 to 7.2)	20.4 (18.3 to 22.5)	−0.2 (−0.3 to −0.1)	−2.3 (−2.7 to −2.0)	7.7 (6.7 to 8.7)
Optimize fluoride levels for suboptimal communities	−4.8 (−6.1 to −3.5)	−15.4 (−17.6 to −13.2)	−0.2 (−0.3 to −0.1)	1.8 (1.5 to 2.1)	−6.6 (−7.7 to −5.5)
10-y Projection					
Removing fluoride	6.1 (4.9 to 7.3)	42.9 (39.8 to 46.0)	−0.5 (−0.6 to −0.3)	−7.6 (−8.4 to −6.8)	15.2 (13.8 to 16.6)
Optimize fluoride levels for suboptimal communities	−5.4 (−6.7 to −4.2)	−34.7 (−38.0 to −31.6)	−0.5 (−0.6 to −0.3)	6.3 (5.6 to 7.1)	−13.9 (−15.4 to −12.4)

Abbreviation: QALYs, quality-adjusted life years.

^a^ Results were obtained from 1000 iterations with Monte Carlo sampling, generating 95% uncertainty intervals from the simulation model.

If all fluoridated areas, including those that are currently suboptimally fluoridated (0.1-0.6 mg/L), received optimal fluoridation levels, the model estimated that dental caries prevalence and total decayed teeth would decrease by 6.9 (95% UI, −8.2 to −5.6) pp and 22.0 million (95% UI, −24.2 to −19.8 million) teeth compared to status quo and save $9.3 billion (95% UI, −10.4 to −8.3 billion) (Table 2). When protective benefits of fluoride were assumed to be applied to only those drinking tap water in fluoridated areas, removing fluoride from PWS had less negative consequences than the base-case scenario; compared to status quo, it was estimated to increase dental caries prevalence and total decayed teeth by 6.0 (95% UI, 4.7 to 7.2) pp and 20.4 million (95% UI, 18.3 to 22.5 million) teeth, respectively, over a 5-year period with costs of $7.7 billion (95% UI, 6.7 to 8.7 billion) (Table 2).

**Figure 2.**
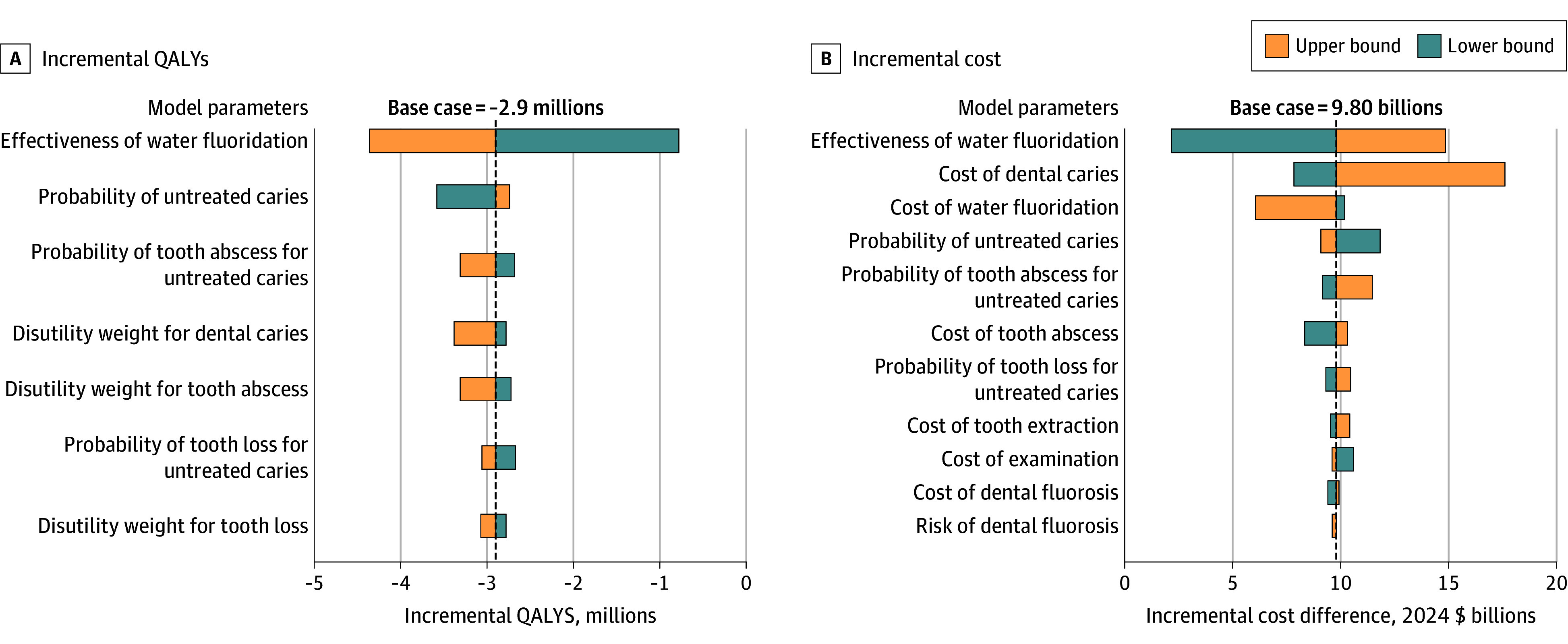
One-Way Sensitivity Analysis Results on Incremental Quality-Adjusted Life Years (QALYs) and Cost Results were obtained from 1000 iterations with Monte Carlo sampling from the simulation model.

None of the sensitivity analyses substantially changed the fundamental findings. In the 1-way sensitivity analysis (Figure 2; eTable 5 in Supplement 1), uncertainty around the effectiveness of water fluoridation in preventing tooth decay was the most influential parameter for both incremental cost and QALYs; even at its lowest efficacy estimate (7.5% reduction in tooth decay vs 25% in the base-case scenario), removing fluoride still estimated to cost $2.08 billion (95% UI, 1.01 to 3.16 billion) and result in 0.76 million (95% UI, −1.09 to −0.43 million) QALYs lost. Dental caries treatment cost was the second most influential parameter for incremental cost. Probability of untreated caries was the second most influential parameter for incremental QALYs.

## Discussion

Our microsimulation estimated that removing fluoride from the PWS would result in greater numbers of dental caries, with associated higher costs to both quality of life and to the health care system. These effects would disproportionately affect publicly insured and uninsured children, who are already at highest risk of unmet dental needs.^46^

Although PWS fluoridation has potential benefits for all individuals, children with reduced access to dental care, such as those from families who are publicly insured, have low income, or live in rural areas, derive the most benefit,^47^ and our model found that removing fluoride would compound disparities in tooth decay. Although all state Medicaid programs are required to cover pediatric dental care, fewer than half of pediatric Medicaid beneficiaries visit a dentist annually.^48^ Although rates of topical fluoride varnish application in the primary care setting (US Preventive Services Task Force−recommended preventive strategy) are higher for publicly insured children and those in rural areas,^49,50^ overall rates hover near 10%, further highlighting the role of PWS as a source of beneficial fluoride exposure for many children.

Our base-case estimates are likely to be conservative because we did not model a benefit to fluoridation for those receiving less than optimal fluoride exposure. Prior work has established a halo effect of living near, but not in, a community with fluoridation of the PWS, which suggests that less than optimal fluoride exposure may still have oral health benefits.^51^ We also did not model the impact of fluoridation on oral health outcomes in adults, although the topical effects of fluoride have a smaller but still present effect on caries rates in adults compared to children^52^ and did not model the potential economic benefit of a healthy dentition.^53^

We did not model a cognitive effect from fluoride exposure. In alignment with current Centers for Disease Control and Prevention and National Toxicology Program recommendations, current levels of fluoride exposure through PWS are not definitively associated with worse neurobehavioral outcomes.^7,13^ PWS are safely fluoridated 99.99% of the time using thresholds set by the Environmental Protection Agency.^23^ It is possible that the mild neurobehavioral changes observed in some, although not all, analyses of prenatal fluoride exposure could have cost implications in adulthood,^54^ yet these effects remain unclear and are beyond the timescale of our simulation. Thus, our analysis was restricted to dental outcomes and their economic implications, rather than all possible theorized health and economic effects of changes in fluoridation of PWS.

### Limitations

Our study has limitations inherent to modeling based on secondary data sources. As forementioned, in the absence of stronger direct evidence (ie, longitudinal observational studies assessing the impact of fluoride on cognitive health outcomes) and current guidance from meta-analyses that the level of fluoridation in PWS is not harmful,^7^ the effects of water fluoridation on the risk of cognitive outcomes were not modeled in our study.

Our baseline simulation population characteristics, such as health risk factors, risk of dental caries, and water fluoridation, informed the NHANES data to generate a nationally representative synthetic population. Because information on water fluoridation levels was collected only for those 0 to 19 years old during 2013 to 2016 in the NHANES, our study sought to simulate the impact of changes in water fluoridation among the child and adolescent population and could not expand our model beyond the current population to minimize uncertainties of the model outputs. Also, we conducted our cost-effectiveness analysis from a health care perspective rather than a societal perspective and focused solely on the costs and benefits of interventions within the health care system itself. Because we did not model the costs and benefits to society of indirect outcomes, such as missed work and school— found to be substantial^55^—our study may provide conservative estimates. Future research should address the impact of changes in water fluoridation on costs and benefits associated with societal outcomes. Next, the data from NHANES, which are subject to the limitations of survey studies, including recall biases, acceptability biases, and underreporting, may lead to underestimation of dental care use; however, because our model estimates the impact of intervention on a relative scale to the baseline, this bias would not change the fundamental findings of this study. Lastly, although uncertainty analyses were performed by sampling from distributions around the input parameter data sources, all possible uncertainties in a simulation model cannot be captured. Probabilistic sensitivity analysis results, generating 95% UIs, may depend on distributional assumptions. Although our distributional assumptions for model parameters were determined to capture overall ranges of the values, the assumed distribution may not resemble true distribution of the parameters and could tend to cluster around the mean, which may affect uncertainty intervals, hence the results are inevitably subject to the assumptions inherent in decision analytic modeling studies. However, to address the issues around distributional assumptions, we conducted 1-way sensitivity analyses with individual model parameters set at their extreme values (lower and upper bounds of the distribution) to evaluate the impact of assuming lowest or highest possible values of the model parameters on the results.

## Conclusions

This cost-effectiveness analysis simulating the results of ceasing PWS fluoridation, per the proposed policy change, projects an increase in tooth decay among children of 7.5 pp and costs of approximately $9.8 billion over 5 years. Subsequent increases in dental costs and disproportionate harms would affect publicly insured and uninsured children. These findings suggest that, despite the potential harms of excessive fluoride exposure, fluoridation at safe levels offers both individual and societal benefits that would be at risk.

## Notes

Supplement 1. eMethods. Model simulation eTable 1. Demographic distribution by water fluoridation level eTable 2. Baseline prevalence of tooth decay eTable 3. Baseline dental utilization eTable 4. Model parameters for one-way sensitivity analysis eTable 5. One-way sensitivity analysis results eFigure. Simulation model validation eReferences.

Supplement 2. Data Sharing Statement
