# Errors in References Cited and Results Section

**DOI:** 10.1001/jamahealthforum.2025.2898

**Published:** 2025-07-11

In the Original Investigation titled “Projected Outcomes of Removing Fluoride From US Public Water Systems”^1^ published online on May 30, 2025, in *JAMA Health Forum*, many of the References in Table 1 were incorrectly cited, a Reference was missing in the Methods section, and a 95% uncertainty interval for costs in the Results section was negative instead of positive. The authors have posted a Comment with the article to explain what happened. This article was corrected online.
