# Supplementary material for: Projected Outcomes of Removing Fluoride From US Public Water Systems
Source: JAMA Health Forum. 2025 May 30;6(5):e251166. doi: 10.1001/jamahealthforum.2025.1166 (PMC12125645; doi:10.1001/jamahealthforum.2025.1166)
Supplement: Supplement 1. — eMethods. Model simulation eTable 1. Demographic distribution by water fluoridation level eTable 2. Baseline prevalence of tooth decay eTable 3. Baseline dental utilization eTable 4. Model parameters for one-way sensitivity analysis eTable 5. One-way sensitivity analysis results eFigure. Simulation model validation eReferences. [file jamahealthforum-e251166-s001.pdf]

Supplemental Online Content

Choi SE, Simon L. Projected outcomes of removing fluoride from US public water systems. *JAMA Health Forum.* Published online May 30, 2025.
doi:10.1001/jamahealthforum.2025.1166

eMethods. Model simulation

eTable 1. Demographic distribution by water fluoridation level

eTable 2. Baseline prevalence of tooth decay

eTable 3. Baseline dental utilization

eTable 4. Model parameters for one-way sensitivity analysis

eTable 5. One-way sensitivity analysis results

eFigure. Simulation model validation

eReferences

This supplemental material has been provided by the authors to give readers additional information about their work.

© 2025 Choi & Simon. *JAMA Health Forum*.

eMethods. Model Simulation

We developed a microsimulation model, which simulates dental care use and risk of tooth decay at the level of the individual. The model is stochastic by sampling from probability distributions of input parameters to generate a distribution of outcomes. The model is run in discrete time steps over the life-course from Dec 2024, where the simulated policy changes are introduced at Dec 2024.

We simulated a nationally representative sample of 10,000 Americans aged 0 to 19 years old starting in Dec 2024 to estimate changes in total costs, quality-adjusted life years (QALYs) and cumulative dental caries incidence with a removal of fluoride in the US public water system accounting for differences in demographic composition and disease risks and access to dental care across the populations residing in areas with different fluoride levels in public water system (eTable 1). We classified synthetic population in this model by combinations of a few key demographic characteristics: age (2-5, 6-12, 13-19 years old), sex, race/ethnicity [National Health and Nutrition Examination Survey (NHANES) categories of non-Hispanic white, non-Hispanic black, Hispanic (Mexican-American or other), and other race], and income [relative to the FPL, adjusted for household size; <130% of the federal poverty level [FPL]), middle (130%-300% of FPL), and high (>300% of FPL)], insurance (private, public, and uninsured), and access to fluoride through the PWS on fluoride concentration levels; [Below detection limit, Less-than-optimal (0.1≤ and ≤0.6 mg/L), Optimal (0.6< and ≤1.5 mg/L), Excessive (> 1.5mg/L)]. Because NHANES is repeated cross-sectional, we had to construct synthetic population to account for the weights. 10,000 individuals were generated, defined by the combinations of these characteristics. The model was re-run 10,000 times while repeatedly Monte Carlo sampling from the probability distributions of model input parameters to capture uncertainties in our estimates.[1]

Baseline dental utilization (annual dental visit) and the risk of tooth decay were modeled and assigned to each simulated individual. The annual risk of dental caries was estimated for each individual as a function of age, race or ethnicity, income level, and insurance status. Annual dental care utilization rate was model as a function of age,

© 2025 Choi & Simon. *JAMA Health Forum*.

sex, income, and insurance status. Binary indicators for caries incidence were assigned to each simulated individual and summed to calculate the total number of decayed teeth for the simulated individuals. To account for individuals aging, we tracked the age of each simulated individual over the simulation period, and updated each individual's probability to utilize dental care and risk of tooth decay to account for their age-specific utilization and health risk by preserving the individual's rank in the population distribution to account for the stability of risk over time. To ensure internal validity, we calibrated the model against dental caries prevalence from NHANES by age groups and race (eFigure). Once individuals develop caries, the probability of caries being treated was 72% based on an analysis of NHANES. For those with untreated caries, the probability of tooth loss was 76.6%, and the probability of tooth abscess was 32.1%.

We used validated equations of age- and sex-specific risk of all-cause mortality as below,

x = age in years

Male: $y = 7e - 6 * e^{0.0773x}$

Female: $y = 4e - 6 * e^{0.0825x}$

© 2025 Choi & Simon. *JAMA Health Forum*.

eTable 1. Demographic distribution by water fluoridation level (proportion)

Total number of children: 79,941,004

	Below detection limit	0.1≤ and ≤0.6 mg/L	0.6 < and ≤1.5 mg/L	> 1.5mg/L
Fluoride level, mean(SD)	0.07 (0)	0.31 (0.02)	0.78 (0.02)	2.05 (0.11)
Overall	0.124	0.457	0.404	0.014
Dental caries	0.436	0.432	0.399	0.503
Age				
0 to 5	0.128	0.450	0.405	0.016
6 to 12	0.123	0.454	0.404	0.018
13 to 19	0.123	0.466	0.401	0.009
Sex				
Male	0.128	0.452	0.404	0.015
Female	0.120	0.463	0.403	0.013
Race/ethnicity				
Hispanic	0.075	0.500	0.389	0.036
NH White	0.150	0.467	0.375	0.007
NH Black	0.092	0.361	0.542	0.006
Other	0.156	0.436	0.400	0.007
Income				
<=130% FPL	0.102	0.442	0.438	0.016
130-300% FPL	0.126	0.494	0.357	0.021
>300% FPL	0.147	0.445	0.400	0.007
Insurance				
Private	0.139	0.467	0.385	0.007
Public	0.111	0.432	0.434	0.023
Uninsured	0.096	0.530	0.356	0.017

*Estimates obtained from NHANES

© 2025 Choi & Simon. *JAMA Health Forum*.

eTable 2. Baseline prevalence of tooth decay

			<6	<6	6 to 12	6 to 12	13 to 19	13 to 19
Sex	Race/ethnicity	Income	Mean	SE	Mean	SE	Mean	SE
Male	Hispanic	Low	0.37	0.06	0.80	0.04	0.65	0.07
		Middle	0.16	0.08	0.65	0.09	0.69	0.09
		High	0.11	0.09	0.63	0.13	0.63	0.13
	NH White	Low	0.34	0.07	0.64	0.06	0.63	0.10
		Middle	0.01	0.01	0.55	0.07	0.72	0.09
		High	0.16	0.06	0.45	0.06	0.57	0.06
	NH Black	Low	0.24	0.04	0.61	0.05	0.53	0.06
		Middle	0.14	0.08	0.59	0.08	0.54	0.09
		High	0.39	0.14	0.68	0.10	0.39	0.08
Female	Hispanic	Low	0.28	0.06	0.77	0.05	0.68	0.06
		Middle	0.27	0.09	0.65	0.08	0.76	0.09
		High	0.08	0.06	0.23	0.08	0.65	0.16
	NH White	Low	0.21	0.07	0.51	0.06	0.78	0.06
		Middle	0.09	0.05	0.39	0.08	0.69	0.09
		High	0.07	0.04	0.45	0.07	0.41	0.07
	NH Black	Low	0.32	0.05	0.57	0.06	0.72	0.05
		Middle	0.23	0.10	0.41	0.08	0.56	0.08
		High	0.01	0.01	0.34	0.10	0.54	0.10

*Estimates obtained from NHANES

© 2025 Choi & Simon. *JAMA Health Forum*.

eTable 3. Baseline Dental Utilization

			<6	<6	6 to 12	6 to 12	13 to 19	13 to 19
Sex	Race/ethnicity	Income	Mean	SE	Mean	SE	Mean	SE
Male	Hispanic	Low	15.65	0.46	40.20	1.84	72.49	2.14
		Middle	13.77	0.76	41.22	2.15	72.21	2.25
		High	14.66	0.77	36.20	3.11	75.35	4.58
	NH White	Low	14.48	0.48	33.71	1.30	73.14	2.36
		Middle	15.01	0.62	37.86	2.08	78.18	4.82
		High	14.80	0.44	36.27	1.23	74.33	2.23
	NH Black	Low	15.36	0.50	35.30	1.25	76.48	2.45
		Middle	14.09	1.35	40.93	2.24	75.23	2.62
		High	15.45	0.90	34.11	2.86	78.35	3.13
Female	Hispanic	Low	14.40	0.38	38.88	1.40	60.63	2.19
		Middle	13.79	0.65	46.19	2.93	65.50	5.13
		High	10.40	0.83	40.34	3.16	59.69	3.65
	NH White	Low	14.00	0.43	40.27	2.37	66.85	2.33
		Middle	14.16	0.53	39.27	1.96	64.12	3.89
		High	14.00	0.53	38.09	1.65	65.52	3.37
	NH Black	Low	15.05	0.53	39.06	1.61	70.35	2.41
		Middle	13.30	0.57	40.37	2.10	67.13	2.03
		High	15.28	0.70	43.35	2.30	68.66	4.17

*Estimates obtained from NHANES

© 2025 Choi & Simon. *JAMA Health Forum*.

eTable 4. Model parameters for one-way sensitivity analysis

Variable	Base-case Value, % or mean (SD	Ranges used in one-way sensitivity analysis	Sources
Disutility weight			
Dental caries**	0.010 (0.003)	(0.0038 - 0.019)	2-3
Tooth abscess	0.069 (0.015)	0.029–0.110	4
Tooth loss	0.067(0.013)	0.045–0.09	3,5
Disease risk			
Effectiveness of water fluoridation*	25%	7.5 to 35%	6,7
Dental fluorosis	7%	7 to 12%	6
Probability of untreated caries	72.0%	45.8-72.0%	8,9
Probability of tooth abscess for untreated caries	32.1%	30.0-46.4%	10-12
Probability of tooth loss for untreated caries	76.6%	66.3-85.5%	13
Cost			
Examination	185 (10)	(45 - 210)	11
Dental caries	530 (20)	(325- 977)	14-16
Abscess	820 (40)	(309 -1220)	17
Tooth extraction	181 (10)	(96 –360)	11
Fluorosis	1460 (420)	(1050-1850)	14-16
Water fluoridation (Annual per capita cost)	0.8 (3.5)	0.6-15.0	18

*A recent Cochrane review[6] found a 4 percentage point difference and 3 percentage point difference for primary and permanent dentition, respectively for caries-free children proportion with community water fluoridation. The 3 percentage point difference would translate to 7.5% reduction in caries (The proportion of caries-free children at base-
line in the non-/low-fluoridated areas was 0.62 – 0.38 with caries. 0.38 to 0.35 reduction in proportions with caries would translate to approximately 7.5% reduction in dental caries, which was used as our lowest effect size for water fluoridation.

**For dental caries disutility weight, base-case value is from the Global Burdens of Disease (GBD).[3] The upper bound of the range came from the upper bound of the disability weights in the GBD study and the lower bound came from Kay et al[2]. Kay et al proposed using acute otitis media (a middle ear infection which also involves acute pain and hospital admissions) as an approximation to calculate the impact of tooth decay when it causes pain, due to the lack of utility estimates for the impact of dental caries from the literature. There were three utility estimates for otitis media (OM): 0.72, 0.79, 0.882 in Kay et al[2]. We used the highest utility weight of 0.882 (corresponding to the lowest disutility weight) in Kay et al[2] to calculate the lower bound for the disutility weight for dental caries. The steps to calculate the lower bound for the child with caries is as follows:

- Utility weight of extraction (estimated from OM): 0.882
- Duration of disutility: 12 weeks
- QALY loss for extraction: (1-0. 882)*(12/52) [difference between disutility of decayed and unerupted tooth, multiplied by the time for which pain/extraction impacted]
- Children with caries who experience acute pain: 13.91%
- Mean QALY loss per child with caries: (1-0. 882)*(12/52) *.1391= 0.0038

© 2025 Choi & Simon. *JAMA Health Forum*.

eTable 5. One-way sensitivity analysis results

Variable	Lower Bound	Incremental QALYs	Incremental Cost	Upper Bound	Incremental QALYs	Incremental Cost
Disease risk						
Effectiveness of water fluoridation	0.075	-0.76 (-1.09,-0.43)	2.08(1.01, 3.16)	0.35	-4.36(-4.69,-4.02)	14.85(13.77, 15.93)
Risk of developing fluorosis	0.07	-2.92 (-3.21, -2.62)	9.57(8.52, 10.62)	0.12	-2.94(-3.25, -2.63)	9.77(8.71, 10.84)
Probability of untreated caries	0.458	-3.58(-3.93, -3.22)	11.83(10.57, 13.10)	0.72	-2.74(-3.08, -2.41)	9.08(8.02, 10.13)
Probability of tooth abscess for untreated caries	0.3	-2.68(-3.01,-2.35)	9.17(8.12, 10.21)	0.464	-3.31(-3.63, -2.99)	11.47(10.31, 12.64)
Probability of tooth loss for untreated caries	0.663	-2.67(-3.01, -2.33)	9.32(8.29, 10.35)	0.855	-3.06(-3.34, -2.77)	10.47(9.39, 11.54)
Cost						
Examination	45	-	10.28(9.22, 11.33)	210	-	9.87(8.79, 10.94)
Extraction	96	-	9.53(8.53, 10.52)	360	-	10.42(9.26, 11.58)
Tooth abscess	309	-	8.33(7.48, 9.18)	1220	-	10.33(9.07, 11.58)
Dental caries	325	-	7.84(6.97, 8.72)	977	-	17.61(15.99, 19.23)
Dental fluorosis	1050	X	9.41(8.37, 10.45)	1850	X	9.93(8.89, 10.97)
Water fluoridation	0.58	-	10.19(9.12, 11.26)	15	-	6.06(5.01, 7.11)
Disutility weight						
Dental caries	0.0038	-2.78(-3.11, -2.46)	-	0.019	-3.38(-3.69, -3.07)	-
Tooth abscess	0.029	-2.89(-3.21, -2.57)	-	0.11	-3.13(-3.45, -2.81)	-
Tooth loss	0.045	-2.78(-3.11, -2.47)	-	0.095	-2.79(-3.11, -2.47)	-

© 2025 Choi & Simon. *JAMA Health Forum.*

eFigure. Simulation model validation

Dental caries (dental caries were calculated based on the number of decayed, missing due to caries, and filled teeth >1). Each colored line represents projected dental caries prevalence from one simulation model iteration. Plots show model outputs from 10,000 iterations with grey shaded area representing the 95% confidence intervals from a Centers for Disease Control and Prevention (CDC) report on dental caries prevalence in the US.

Overall prevalence

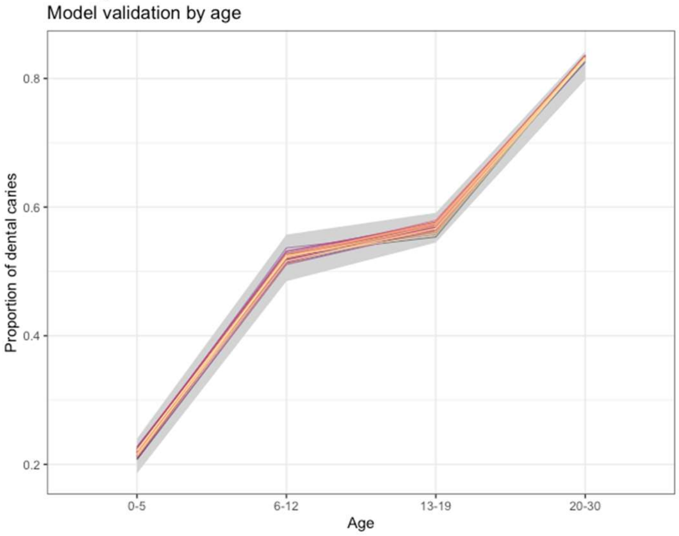

Prevalence by age among Hispanic

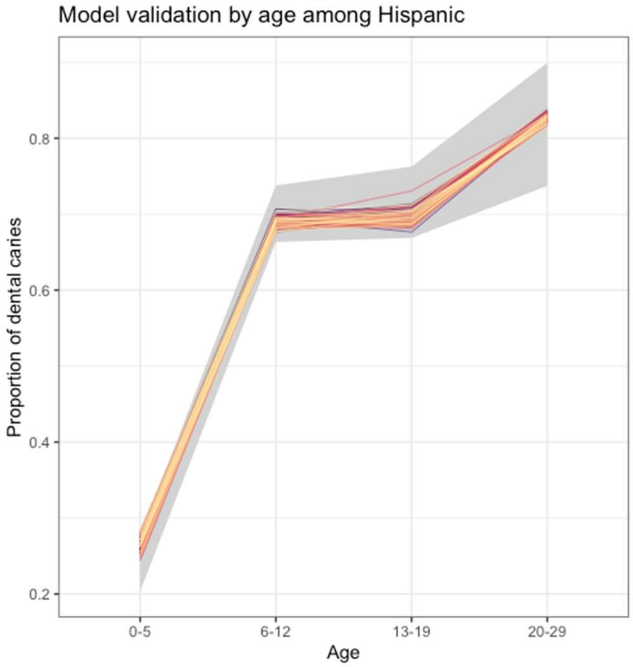

Prevalence by age among Non-Hispanic White

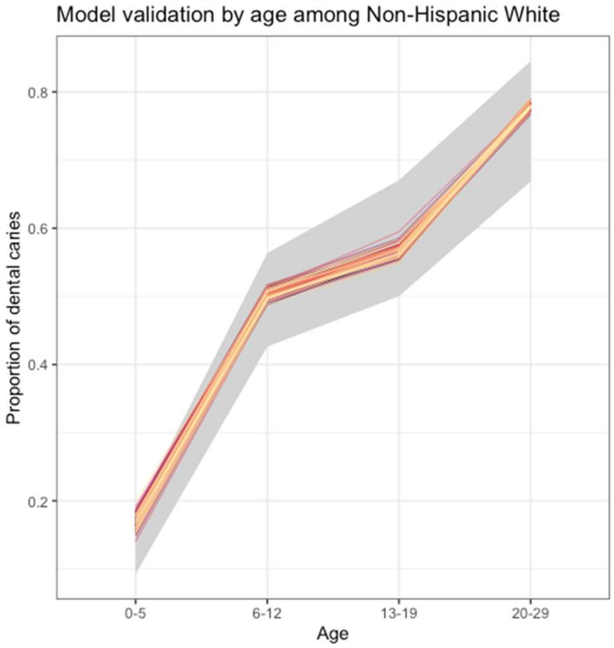

© 2025 Choi & Simon. *JAMA Health Forum*.

Prevalence by age among Non-Hispanic Black

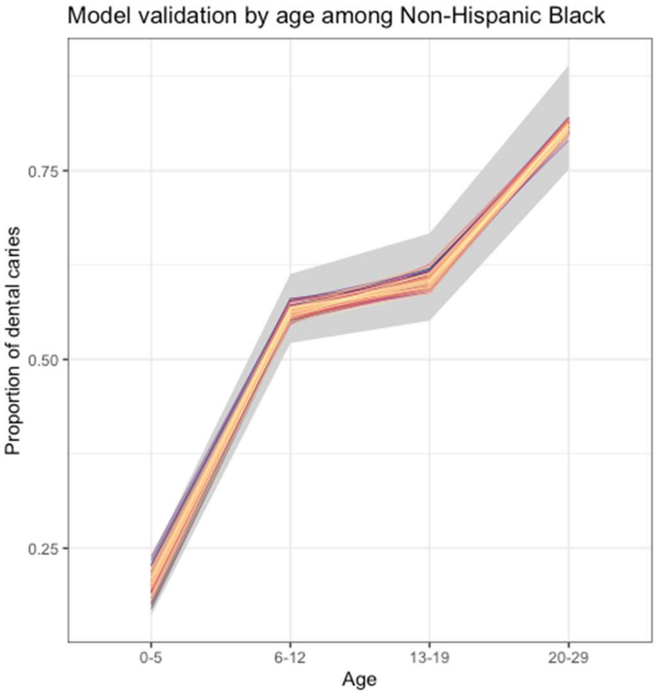

Prevalence by age among Other race

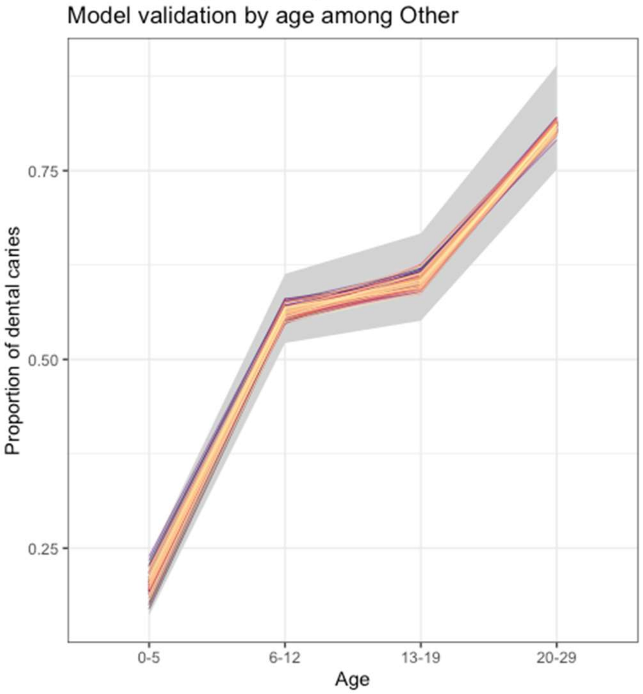

© 2025 Choi & Simon. *JAMA Health Forum*.

eReferences

1. Ramsey S, Willke R, Briggs A, et al. Good research practices for cost-effectiveness analysis alongside clinical trials: the ISPOR RCT-CEA Task Force report. Consensus Development Conference
Research Support, Non-U.S. Gov't
Review. *Value Health*. Sep-Oct 2005;8(5):521-33. doi:10.1111/j.1524-4733.2005.00045.x
2. Kay E, Owen L, Taylor M, Claxton L, Sheppard L. The use of cost-utility analysis for the evaluation of caries prevention: an exploratory case study of two community-based public health interventions in a high-risk population in the UK. *Community Dent Health*. Mar 1 2018;35(1):30-36. doi:10.1922/CDH_4115Owen07
3. Institute for Health Metrics and Evaluation (IHME). Global Burden of Disease Study 2019 (GBD 2019) Disability Weights. Accessed Jan 2, 2022. https://ghdx.healthdata.org/record/ihme-data/gbd-2019-disability-weights#:~:text=Disability%20weights%2C%20which%20represent%20the,health%20and%201%20equals%20death.
4. Brennan DS, Spencer AJ. Disability weights for the burden of oral disease in South Australia. *Popul Health Metr*. Sep 3 2004;2(1):7. doi:10.1186/1478-7954-2-7
5. Evaluation IfHMa. Oral disorders. Accessed Nov 11, 2024. https://www.healthdata.org/sites/default/files/methods_appendices/2021/Oral_nonfatal_2020_final_jw_updated_atm_Apr_17_AC.pdf
6. Iheozor-Ejiofor Z, Walsh T, Lewis SR, et al. Water fluoridation for the prevention of dental caries. *The Cochrane database of systematic reviews*. Oct 4 2024;10(10):CD010856. doi:10.1002/14651858.CD010856.pub3
7. Boehmer TJ, Lesaja S, Espinoza L, Ladva CN. Community Water Fluoridation Levels To Promote Effectiveness and Safety in Oral Health - United States, 2016-2021. *MMWR Morb Mortal Wkly Rep*. Jun 2 2023;72(22):593-596. doi:10.15585/mmwr.mm7222a1
8. Fleming E, Afful J. Prevalence of Total and Untreated Dental Caries Among Youth: United States, 2015-2016. *NCHS Data Brief*. Apr 2018;(307):1-8.
9. Centers for Disease Control and Prevention. Prevalence of Total and Untreated Dental Caries Among Youth: United States, 2015–2016. Accessed May 5, 2024. https://www.cdc.gov/nchs/products/databriefs/db307.htm
10. Azodo CC, Chukwumah NM, Ezeja EB. Dentoalveolar abscess among children attending a dental clinic in Nigeria. *Odontostomatol Trop*. Sep 2012;35(139):41-6.
11. Srivastava VK. Prevalence of Abscesses Associated with Carious Primary Teeth in Preschool Children and its Association with Age, Gender, Location, and Parent's Education and Social Class: An Observational Study. *Int J Clin Pediatr Dent*. May-Jun 2022;15(3):287-292. doi:10.5005/jp-journals-10005-2376
12. Schnabl D, Fleischer F, Riedmann M, Laimer J, Gassner R. Prevalence and distribution of deep caries and abscess formation in children who required emergency dental general anaesthesia. A retrospective analysis. *Eur J Paediatr Dent*. Jun 2019;20(2):119-122. doi:10.23804/ejpd.2019.20.02.07
13. Monte-Santo AS, Viana SVC, Moreira KMS, Imparato JCP, Mendes FM, Bonini G. Prevalence of early loss of primary molar and its impact in schoolchildren's quality of life. *Int J Paediatr Dent*. Nov 2018;28(6):595-601. doi:10.1111/ipd.12416

© 2025 Choi & Simon. *JAMA Health Forum*.

14. American Dental Association. Survey of Dental Fees. Accessed Feb 13, 2019. https://success.ada.org/en/practice-management/finances/survey-of-dental-fees
15. Atkins CY, Thomas TK, Lenaker D, Day GM, Hennessy TW, Meltzer MI. Cost-effectiveness of preventing dental caries and full mouth dental reconstructions among Alaska Native children in the Yukon-Kuskokwim delta region of Alaska. *J Public Health Dent*. Jun 2016;76(3):228-40. doi:10.1111/jphd.12141
16. Humana. Cost of common dental procedures. Accessed Jan 5, 2022. https://www.humana.com/dental-insurance/dental-resources/cost-of-dental-procedures
17. UnitedHealthcare. Dental Fee Schedule. Accessed May 15, 2022. https://vaccnprovidertemplate.logisticshealth.com/trainingdocs/VACCN-ProvTrng-DentalFeeSchedule.pdf
18. O'Connell J, Rockell J, Ouellet J, Tomar SL, Maas W. Costs And Savings Associated With Community Water Fluoridation In The United States. *Health Aff (Millwood)*. Dec 1 2016;35(12):2224-2232. doi:10.1377/hlthaff.2016.0881

© 2025 Choi & Simon. *JAMA Health Forum*.
